# Severe Relapsing Clozapine-Withdrawal Catatonia

**DOI:** 10.1155/2015/606853

**Published:** 2015-12-14

**Authors:** Tarek Shahrour, Muez Siddiq, Saad Ghalib, Taoufik Alsaadi

**Affiliations:** ^1^Department of Psychiatry, Sheikh Khalifa Medical City, P.O. Box 51900, Abu Dhabi, UAE; ^2^Department of Neurology, Sheikh Khalifa Medical City, P.O. Box 51900, Abu Dhabi, UAE

## Abstract

Catatonia as a clozapine-withdrawal syndrome has only been documented in the medical literature as case reports. We are reporting a case in which a 32-year-old man develops a catatonic state upon withdrawal of clozapine. The state was quite severe and needed ICU admission. The course was chronic and intermittent which we think was caused by the poor adherence to antipsychotics. The importance of identifying such cases early is underlined.

## 1. Introduction

The emergence of catatonia upon abrupt discontinuation of clozapine has been reported in the medical literature mainly as case reports [1]. Therefore this area remains greatly understudied and speculative.

In the following case, we wish to emphasize that such clinical picture may be difficult to diagnose, can necessitate dramatic intervention (e.g., ICU admission, Intubation, and Mechanical ventilation), and, moreover, can have a chronic relapsing course, if not appropriately diagnosed and managed.

## 2. Case Report

We present a 32-year-old unemployed, single Yemeni national with 10-year history of schizophrenia characterized by symptoms of auditory hallucinations and passivity phenomenon who was evaluated for change of mental status. He was stable for nearly 6 years on a combination of clozapine 400 mgs/day and olanzapine 10 mgs/day. There were no catatonic features in his previous history.

In March 2014, he presented to outpatient department with anxiety, tremor, dizziness, and history of a fall, which was accompanied by slowness in movement. Medication was stopped as this was thought to be medication side effects but in hindsight it was more likely to be withdrawal symptoms rather than the opposite. A physical work-up identified no abnormalities. He was subsequently resumed on olanzapine, to which, he was partially adherent, but defaulted from the service for four weeks and as a result was out of medication. Four weeks later, he presented to the emergency room with altered mental status and generalized jerky movements characterized by fast rhythm tremors in the four limbs, associated with deterioration in the level of consciousness (GCS 5). He was intubated and admitted to the ICU. Status epilepticus was suspected with herpes encephalitis as a possible differential. NMS was also considered but the treating team was not sure if he was taking his antipsychotics, which was olanzapine at the time. He had a full medical work-up including EEG, LP, and brain MRI, which were all found to be within normal limits. He was treated with phenytoin and given an empirical course of acyclovir. He showed some improvement and the family discharged him against medical advice 6 days later on levetiracetam 1000 mg/a day. The fact that he was out of medication would suggest that this particular presentation was probably related to withdrawal from medication.

In the ensuing few months, the patient presented several times to the ER, with altered mental status and again fast rhythm tremors in the four limbs, talking and repeating few words irrelevantly (perseveration), repeating the words of the examiner (echolalia), and staring in an odd manner. Because of the delirium-like picture in each and every presentation, a full medical work-up was arranged, and a plan was put in place to restart him on olanzapine and lorazepam once his case was cleared medically. However, the patient's family has prematurely discharged him against medical advice. The adherence between admissions was quite erratic. In each of these presentations, the patient stayed in the hospital from few hours up to 3 days maximum.

On his most recent admission (December, 2014), both the neurology and psychiatry teams have reviewed his medical records in detail. A full neurological work-up was unremarkable. Toxicology screen was negative. He had a prolonged video EEG that did not show any ictal EEG changes during the time he was having the same tremor-like movements and the other catatonic symptoms. In addition, no interictal epileptiform discharges were seen. The main provisional diagnosis was that of catatonia and he was managed with IV lorazepam 8 mgs a day. He showed a mild improvement and was later transferred to the psychiatric ward following extensive family meetings. His AEDs were gradually reduced with a plan to have them eventually stopped.

He was treated with olanzapine, lorazepam, procyclidine, and levetiracetam (which was to be gradually stopped) and he made significant improvement. He was discharged home 3 weeks following admission to the psychiatry ward with no active psychotic symptoms, no movement disorder, and major improvement in his speech and thought form. Clozapine was not reinstated due to patients' erratic adherence in the past.

## 3. Discussion

Our case, similar to the very few reported cases of catatonia induced by withdrawal of clozapine, suggests that our patient's relapse was due to an abrupt discontinuation of clozapine but the nature of his relapse was catatonic, which is noticeably different from his original clinical picture. This latter observation is in agreement with other case reports in the literature [2]. The catatonic picture was, at one stage very severe, where he had a delirium-like picture and needed an ICU admission. This would suggest that the type of catatonia our patient had was of the malignant type (lethal).

We would like to point out that the diagnosis was delayed by several factors, including the time needed to exclude medical reasons, the lack of cooperation from family, and the lack of adherence to medication, which most probably led to the aforementioned chronic relapsing picture. Epileptic seizures were excluded as he had negative vEEG recording whilst in the active stage with the reported movement disorder fully recorded and analyzed.

On the other hand, our case differs from the other similar reported cases in that the patient was off clozapine for weeks before presenting with the full-blown picture of catatonia. Of course it can be argued that this was an independent relapse. However, the temporal relationship and the new catatonic picture would suggest otherwise. The delayed presentation can partially be explained by the intermittent exposure and withdrawal of olanzapine after the discontinuation of clozapine. Still, our case strengthens the idea that Moncrieff presented in her paper [3] where she argued that the illness that follows the discontinuation of an antipsychotic could have nothing to do with the original psychotic illness.

Indeed, up to 50% of patients on clozapine discontinue their treatment mainly, due to side effects and the need to have regular blood tests [4]. This case report would highlight the issue of nonadherence, as one of the main difficulties when dealing with clozapine treatment. Clozapine is recognized as one of the main culprits in causing rebound psychosis, due to its wide spectrum effects on brain receptors. In our case, prescribing different antipsychotic to clozapine along with an anticholinergic agent was an effective strategy, when adhered to by the patient resulting in good recovery, although it took longer than what was previously reported in the literature. This case report would further strengthen the notion that response to benzodiazepine in malignant catatonia is not as dramatic as the response of simple catatonia and that a lorazepam test is not sensitive enough in these cases [5].

A multidisciplinary approach is always vital to ensure the involvement of patient and caregivers earlier on. A practical suggestion is to prescribe depot antipsychotic with a high anticholinergic potency alongside clozapine in patients with history of erratic adherence.
